# Supplementary material for: Immunomodulatory potential of Clinacanthus nutans extracts in the co-culture of triple-negative breast cancer cells, MDA-MB-231, and THP-1 macrophages
Source: PLoS One. 2021 Aug 11;16(8):e0256012. doi: 10.1371/journal.pone.0256012 (PMC8357171; doi:10.1371/journal.pone.0256012)
Supplement: Attachment — Submitted filename: Review.docx [file pone.0256012.s001.docx]

Triple negative breast cancer is a difficult to treat phenotype that definitely warrants further investigation. Research into natural ‘local’ remedies to underpin the scientific basis for any effects is interesting and this paper aims to link the two fields by investigating the effects of a well known SE Asian medicinal plant *C.Nutans* on a triple negative breast cancer cell line and a macrophage cell line *in vitro*.

Crude extracts from plants contain many phytochemicals, as indicated by the qualitative observation of a wide range of constituents (table 1 ie containing Saponin, Alkaloid, Phenol and Tannin, Flavonoid, Terpenoid, Glycoside, Steroid). HPLC demonstrated a number of flavones and one flavonoid (schaftoside) in the ethanol extract, as may be expected. It isn’t possible to evaluate what the active components are, and whether they could be reproduced by a single purified entity (eg after full characterization by spectral techniques (eg MS, NMR, IR, etc). I am slightly unclear whether this manuscript is about drug discovery (potentially novel compounds from the plant) or herbal medicine, since both are mentioned.

The ethanol and aqueous extracts did not impact on cell viability, and the concept of cancer as a proliferative disease is now old fashioned in the era of immunotherapy. However the authors make a valid point that only screening for agents which inhibit the proliferation of tumour cell lines will miss compounds that effect different pathways. The scratch assay indicated that the extracts did not effect migration either.

It was interesting that the authors included a co-culture experiment to evaluate the effects of the extract on cytokine production. THP-1 are a monocytic leukemia cell line and were differentiated to macrophages using PMA. However, this does not mean they have the characteristics of M2 macrophages (as indicated in the discussion). Other authors consider THP-1 as a good model of M0/M1 differentiation and there are publications using IL4 and IL13 to drive M2 differentiation eg Genin, M., Clement, F., Fattaccioli, A. et al. M1 and M2 macrophages derived from THP-1 cells differentially modulate the response of cancer cells to etoposide. BMC Cancer 15, 577 (2015). <https://doi.org/10.1186/s12885-015-1546-9>

I think more specific detail on the co-culture model would be valuable, the seeding density of THP-1 on inserts was stated, but the seeding density / confluence of MDA-MB231 triple negative breast cancer cell line was not (just that they were cultured for 6 days). Was the extract (1h in serum free medium) placed in the upper chamber, lower chamber or both? The differentiated THP-1 were then primed with LPS (20ng/ml), to stimulate cytokine production – which is more representative of infection and not cancer. 18h later the culture supernatant was removed for cytokine analyses – was this the upper chamber / lower chamber and do the authors thing the cytokine was from the macrophages only or there could be any contribution by the epithelial cell line? What is the rationale for including the epithelial cells in the co-culture experiments – what contribution do the authors think they are making?

The cytokine data was presented as % of activation rather than the absolute values. I think it would be interesting to see the amount of cytokine produced by LPS stimulated THP-1 +/- extracts. It isn’t clear in the methods how many replicates and whether the data produced is normally distributed. If it is, then ANOVA is more appropriate than a simple t test.

Ultimately, the authors state that their extracts are anti-inflammatory on the basis of the changes in production of IL6, IL1β and TNFα, although the aqueous extract seems to show a slight increase in IL6 and TNFα (above 100%). If there was a meaningful effect, then a dose response should be seen (not evident for IL6 and IL-1β with the ethanol extract).
