# Supplementary material for: Immunomodulatory potential of Clinacanthus nutans extracts in the co-culture of triple-negative breast cancer cells, MDA-MB-231, and THP-1 macrophages
Source: PLoS One. 2021 Aug 11;16(8):e0256012. doi: 10.1371/journal.pone.0256012 (PMC8357171; doi:10.1371/journal.pone.0256012)
Supplement: Attachment — Submitted filename: Response to Reviewers.docx [file pone.0256012.s003.docx]

Immunomodulatory potential of *Clinacanthus nutans* extracts in the co-culture of triple-negative breast cancer cells, MDA-MB-231, and THP-1 macrophages.

*Nor Fadilah Rajab, Biomedical Science Program, Center for Healthy Aging and Wellness, Faculty of Health Sciences, Universiti Kebangsaan Malaysia, Kuala Lumpur, Malaysia.* [*nfadilah@ukm.edu.my*](mailto:nfadilah@ukm.edu.my)

Dear Editor,

I am writing with regards of the manuscript submission (Manuscript ID: PONE-D-20-27749). Enclosed are the notes for all reviewer’s comments and revised manuscript for your perusal.

We hope that the we have addressed all comments accordingly and the manuscript will be given a fair consideration for submission. I am looking forward to hearing from you soon.

Thank you.

Yours Sincerely,

Prof. Dr Nor Fadilah Rajab

Deputy Dean (Research and Innovation).

Faculty of Health Sciences,

Universiti Kebangsaan Malaysia

Jalan Raja Muda Abd Aziz,

50300 Kuala Lumpur

**Appendix**

Notes for reviewer’s comments are as below and included in the uploaded revised manuscript.

**Reviewer #1:**

1. Please check all reference before final submission. It is also recommended to avoid any literature that is retracted.

- Authors have checked all references and make sure that there is no retracted literature included in the manuscript to date. Five references have been removed and 2 newly added references has been updated in the list.

**Reviewer #2:**

1. CN is an interesting plant and it’s ethnobotanic historic uses certainly recommend further investigation of it’s medicinal properties.

The authors isolate phytochemicals from CN leaves using ethanol / aqueous methods and partially characterize their contents using a range of qualitative tests plus HPLC. The extracts are not cytotoxic and do not induce migration in a scratch assay.

It is an interesting observation that 1h pre-treatment with CN extracts can affect LPS-induced pro-inflammatory cytokine production from human cancer cell lines ie THP-1 macrophage / MDA-MB-321 epithelial co cultures in vitro.

- Yes. Similar observations can be obtained from the study by Mai et al. 2016 (<https://dx.doi.org/10.3389%2Ffphar.2016.00007>). They showed that an hour pre-treatment with CN extract can affect the pro-inflammatory cytokines expression in mouse macrophages RAW 264.7 cells.

2. The authors demonstrated that ethanol extracts decreased IL6 production at 25µg/ml and 100µg/ml but not 50µg/ml, however IL6 levels were significantly *increased* with 50µg/ml and 100 µg/ml of aqueous extracts. A similar mixed pattern was seen with IL-1β production in response to the ethanol extracts (ie decreased at 25µg/ml and 100µg/ml but not 50µg/ml). Aqueous extracts inhibited IL-1β production at the two lower concentrations. CN extracts (both ethanolic and aqueous) significantly inhibited TNF alpha production at all the concentrations tested. I presume that the assay conditions were optimized, but it would be interesting to see dose responses over time. Both cell lines are known to express TLR4, which I guess justifies the use of LPS in this model. I am not sure 3 data points (low, high, low) can be reliably called ‘biphasic’ without further information.

- Authors have removed the biphasic effect from the discussion part. The corrections have been included in the revised manuscript.

“Our results showed that 25 and 100 µg/mL ethanolic CN extract suppressed the secretion of IL-6 and IL-1β in the co-culture between human TNBC cell line, MDA-MB-231 and human macrophage-like cells such as THP-1 (Figure 7a and 7b). By contrast, CN aqueous extract increased the secretion of IL-6 at 50 and 100 µg/mL but not of IL-1β at 25 and 50 µg/mL. LPS-induced production of pro-inflammatory cytokines were regulated by multiple pathways such as Nrf2/Keap1 and NF-κB pathways thus overlapping modulation of these pathways may have occurred at certain CN concentration as previously shown by other plant phytochemicals [62]. Other than that, the contribution of CN antioxidant effect may have altered the pro-inflammatory cytokine milieu in the co-culture [63,64]. High IL-6 expression in co-culture experiment may be due to the high basal IL-6 secretion from MDA-MB-231 cells besides production of LPS-induced pro-inflammatory cytokines from THP-1 macrophages [65]. These results suggested that CN extracts were able to ameliorate the inflammation state in the TME via inhibition of IL-6 or IL-1β secretions from the cancer cells and immune cell interactions at certain concentrations.”

3. Whilst it is good to mimic a tumor micro-environment in vitro, care has to be taken when extrapolating findings and this manuscript has gone too far in interpreting these observations as representing an 'anti-cancer' effect. I disagree that cultured THP-1 represent M2 macrophages, and they are considered more like M1 in the literature (as previously stated).

- Authors agreed to amend the interpretation of the results. Instead of emphasizing on the anticancer effect, we rewrite the discussion part to suggest that CN ameliorates the inflammation condition between cancer cells and macrophages. The appropriate corrections have been included in the revised manuscript.

**Reviewer #3:**

The article by Nordin et al evaluates the effects of Clinacanthus nutans on the co-culture of MDA-MB-231 cancer cells and human THP-1 macrophages, by looking at its effects on the viability, migratory and inflammatory milieu of the co-culture.

The manuscript is well-written and conclusions are drawn appropriately given the supporting evidence.

The authors have responded appropriately to the recommended revisions given by the previous two reviewers.

Minor comments:

Introduction section “the leaves of this plant are commonly used as water decoction for oral ingestion or soaked in alcohol for topical application to the affected area [9]”

‘the affected area’ should have been clarified.

- Authors decided to amend the phrase, “soaked in alcohol for topical application to the affected area [9]” as it is related to herpes related diseases remedy. We rephrased the statement as “As anticancer remedies, the leaves of this plant are commonly used as water decoction for oral ingestion”.
